# Genome rearrangements as double coset Markov chains

**DOI:** 10.1007/s00285-026-02450-x

**Published:** 2026-07-27

**Authors:** Mackenzie A. Simper

**Affiliations:** School of Medicine, Washington University in St. Louis, 660 S. Euclid Ave., St. Louis, Missouri 63110 USA

**Keywords:** Genome rearrangement, Double coset, Markov chain, Hyperoctahedral group, 20B35, 60J10, 92D15, 05A05

## Abstract

Permutation groups provide a natural framework for modeling genomes and their rearrangement events, which is crucial for understanding evolutionary relationships. Double cosets can be used to model objects arising from multiple symmetries, such as circular genomes with repeated genes. Suppose ${\boldsymbol{\lambda } = \boldsymbol{( \lambda _1,}\boldsymbol{\lambda _2,} \boldsymbol{\dots , \lambda _k)}}$ is a partition of ${{\boldsymbol{n}}}$ which indicates the number of repeated regions or genes of different types ($\boldsymbol{\lambda _i}$ of type ${{\boldsymbol{i}}}$ with ${{\boldsymbol{n}}}$ total regions). A circular genome with ${{\boldsymbol{n}}}$ oriented regions determined by $\boldsymbol{\lambda }$ can be identified with the double coset space ${\boldsymbol{S}}_{\boldsymbol{\lambda }} \backslash {\boldsymbol{B}}_{\boldsymbol{n}} / {\boldsymbol{D}}_{\boldsymbol{n}}$, where $\boldsymbol{B_n}$ is the hyperoctahedral group, $\boldsymbol{D_n}$ the dihedral group extended to $\boldsymbol{B_n}$, and $\boldsymbol{S_\lambda }$ the Young subgroup extended to $\boldsymbol{B_n}$. This paper develops this correspondence and derives formulas for the sizes of double cosets and the number of double cosets in special cases. The size of double cosets gives the induced probability distribution on genomes from uniformly sampling $\boldsymbol{B_n}$. The representation is utilized to define Markov chains which capture the processes of inversions, transpositions, and translocations.

## Introduction

Genome rearrangements—such as inversions, translocations, duplications, and deletions—are a primary source of structural and biological variation between species. These events have long been studied as a way to measure evolutionary distance between genomes, beginning with early observations of inversions in *drosophila* (Sturtevant and Dobzhansky 1936) and rearrangements in mitochondrial genomes in plants (Palmer and Herbon 1988; Bafna and Pevzner 1995). The basic idea is to define some sort of ‘rearrangement distance’ between genomes which can be used as a proxy for evolutionary distance. A pairwise distance can then be input into phylogenetic algorithms for tree reconstruction e.g., (Saitou and Nei, 1987; Moshe et al., 2022). Classical algorithmic work focused on reversal events (Bafna and Pevzner 1996; Hannenhalli and Pevzner 1999) and was later generalized to more complex operations such as the double cut and join model (Yancopoulos et al. 2005; Bergeron et al. 2006).

An algebraic approach views genomes as elements of permutation groups, where rearrangement operations correspond to group actions. This perspective has proven powerful in the study of inversions and related operations (Francis 2014; Egri-Nagy et al. 2014; Sumner et al. 2017; Terauds and Sumner 2022; Stevenson et al. 2023). However, the basic permutation group framework assumes all genes are distinct, while in reality genomes may contain repeated elements due to duplications or horizontal gene transfer (especially common in plant genetics, see e.g. (Bader 2010; Sankoff 2001)). A richer model is therefore required to account for both symmetries and repetitions. In this paper, we show that double cosets provide a natural algebraic model for circular genomes with repeated elements.

***Double cosets for genomes*** If *G* is a finite group and *H*, *K* are two subgroups of *G*, then the H\G/K *double cosets* are the equivalence classes defined by the equivalence relation

$$ s \sim t \iff s = h t k \,\,\,\,\,\,\,\, \text {for} \,\,\,\,\,\,\,\, s, t \in G, \,\,\, h \in H, \,\,\, k \in K. $$

Recently, double cosets have been studied due to their interesting combinatorial properties and as a framework for defining random processes (Diaconis et al. 2023; Simper 2022; Schwob 2025; Diaconis et al. 2026). A fundamental question is: What is the distribution on H\G/K induced by picking a element uniformly from *G* and mapping to its double coset? This is equivalent to counting the size of each equivalence class. Additionally, Markov chains on the base group *G* can induce Markov chains on the double coset space. Recent work (Britnell and Wildon 2024; Simper 2022) answers the question: Given a Markov chain on *G*, is the lumped process on H\G/K also a Markov chain?

Double cosets have been previously used to model genome rearrangement problems (Stevenson et al. 2023), though not in the context of repeated elements. The hyperoctahedral group $B_n$ is the starting point to consider a linear genome with genes or segments of genes which have an orientation. That is, the genome has a fixed reading direction, with a distinguished first/last gene and each gene also has an orientation; this models, for example, a linear chromosome or a DNA sequence with a defined 5’ to 3’ orientation. A circular genome, in contrast, has no fixed start or end (any rotation produces the same biological arrangement) and is the appropriate model for many bacterial chromosomes and plasmids. If $D_n$ is the dihedral group (symmetries of an *n*-gon) which is extended to be a subgroup of $B_n$, then the left cosets $B_n / D_n$ identify circular genomes with an orientation. Suppose $\lambda = (\lambda _1, \dots , \lambda _k)$ is a partition of *n*, (that is, $\lambda _1 \ge \lambda _2 \ldots \ge \lambda _k > 0$ are integers with $\sum _i \lambda _i = n$). Then λ can signify the number of repeated genes of the same type, i.e. $\lambda _1$ occurrences of type 1, $\lambda _2$ of type 2, and so on. The Young subgroup $S_\lambda \cong S_{\lambda _1} \times S_{\lambda _2} \times \ldots \times S_{\lambda _k}$ consists of permutations which interchange only genes of the same type with one another. The double coset space $S_\lambda \backslash B_n / D_n$ identifies circular genomes with *n* oriented regions and repeated regions according to λ. Equivalently, $S_\lambda \backslash B_n / D_n$ is in bijection with the orbits of $S_\lambda$ acting by left multiplication on the coset space $B_n / D_n$; two oriented circular genomes are in the same double coset if and only if one can be obtained from the other by relabeling repeated genes.

The main result in this paper, Theorem 1, counts the size of each double coset and thus identifies the probability distribution induced by sampling uniformly from $B_n$ and mapping to genomes. We also apply double coset Markov chain theory to define several possible Markov chains which model biological mutations and have stationary distribution determined by Theorem 1. The uniform distribution on $B_n$ is a natural statistical baseline as the maximum-entropy distribution over all signed permutations, with no assumption about which gene orders or orientations are preferred. It also represents the long-run equilibrium of any unbiased random rearrangement process on the base group, since any ergodic random walk on $B_n$ converges to the uniform distribution. Genome configurations with more rotational or reflective symmetry will correspond to larger double cosets and are therefore more probable under this baseline.

***Outline*** Section 2 introduces the group theory background and notation before carefully describing the double coset space $S_\lambda \backslash B_n / D_n$ and how double cosets can be visualized as circular genomes with repeated elements. We find the induced distribution on double cosets from the uniform distribution on $B_n$. Section 3 introduces some possible moves for transitioning between genomes, and specific random walks on $B_n$ which induce Markov chains on the double cosets. The ‘flip-transpose’ Markov chain has known eigenvalues on $B_n$ which can be used to give an upper bound on the rate of convergence. The final section suggests some open problems.

## The double coset space

In this section, we set the notation and conventions for working within the symmetric group and define the hyperoctahedral group, dihedral group, and Young subgroup. Then we discuss the double coset space and compute a formula for the induced distribution. See Bhatia et al. (2018) for a careful review of the different ways permutations have been used to represent genomes.

### Group theory background

***Symmetric group*** The symmetric group $S_n$ is the set of all permutations of *n* unique symbols. We will assume each symbol represents an unique ‘gene’ (or ‘synteny block’) within a genome or chromosome. A permutation records the order in which the genes are arranged (following the *position paradigm* as described in (Bhatia et al. 2018)). In *two-line notation*, an example $\sigma \in S_n$ is

$$ \sigma = \begin{pmatrix} 1 & 2 & 3 & 4 & 5 \\ 2 & 3 & 1 & 5 & 4 \end{pmatrix}: \quad \quad \quad \begin{pmatrix} \text {position} \\ \text {symbol} \end{pmatrix}. $$

This σ indicates the permutation for which the gene labeled 2 is in the first position, 3 is in the second position, and so on. (Equivalently, one can think of the first row as the labels of the genes and the second row the positions.) The *one-line notation* (which we will use most frequently) is simply the second row σ=23145.

Sometimes it will be helpful to write $\sigma \in S_n$ using *cycle notation*:

$$\begin{aligned} \sigma = 23154 = (123)(45). \end{aligned}$$

For example, the cycle (123) can be read as ‘position 1 maps to label 2, position 2 maps to label 3, position 3 maps to label 1’. We can also notate σ as a function mapping positions to symbol and write σ(i) for the symbol at position *i* and $\sigma ^{-1}(i)$ for the position of the symbol labeled *i* (here σ(3)=1 and $\sigma ^{-1}(3) = 2$).

Multiplication of permutations will be calculated right-to-left. That is $(\rho \cdot \sigma )(i) = \rho (\sigma (i))$. For example, if ρ=13425=(234), then

$$\begin{aligned}&\rho \cdot \sigma = 13425 \cdot 23154 = 34152 \\&\sigma \cdot \rho = 23154 \cdot 13425 = 21534 \end{aligned}$$

Multiplication on the left by a permutation σ then corresponds to applying σ to the labels; multiplying on the right corresponds to applying σ to the positions. For instance, a *transposition* is a permutation with cycle structure τ=(ij)=(i,j). Multiplication on the left by τ then exchanges the symbols *i*, *j*, e.g. $(23)\cdot \rho = 12435$, and multiplication on the right exchanges the positions *i*, *j*. e.g. $\rho \cdot (23) = 14325$.

***Hyperoctahedral group*** The symmetric group $S_n$ can represent linear genomes with fixed orientation: that is, there is a distinguished first gene (head) and last gene (tail), corresponding biologically to genetic sequences with a specific reading order. The labels 1,2,⋯,n denote distinct genes (or regions) in the genome. We can expand the notion to account for orientation of the individual genes using the hyperoctahedral group, defined as $B_n \cong C_2^n \rtimes S_n$. Here $C_2^n = \{+1,-1\}^n$ is the group of sign changes on *n* coordinates and $S_n$ acts on $C_2^n$ by permuting coordinates. The semidirect product $C_2^n \rtimes S_n$ consists of pairs $(\boldsymbol{\epsilon }, \sigma )$ with $\boldsymbol{\epsilon } \in C_2^n$ and $\sigma \in S_n$, multiplied by the rule $(\boldsymbol{\epsilon },\, \sigma )\cdot (\boldsymbol{\epsilon }',\, \sigma ') = \bigl (\boldsymbol{\epsilon }\cdot \sigma (\boldsymbol{\epsilon }'),\; \sigma \sigma '\bigr ),$ where $\sigma (\boldsymbol{\epsilon }')$ permutes the components of $\boldsymbol{\epsilon }'$ according to σ. Thus, $|B_n| = n! \cdot 2^n$ and we can think of $B_n$ as the set of *signed permutations* of *n*, i.e. every symbol has one of two possible orientations. We can also see $B_n \subset S_{2n}$ as the subgroup of centrally symmetric permutations: $\sigma \in S_{2n}$ with $\sigma (i) + \sigma (2n + 1 - i) = 2n + 1$ for all $1 \le i \le n$. For example, when n=2 we have $|B_2| = 8$ and, as elements of $S_4$, can write

$$ B_{2} = \{1234, 4231, 1324, 4321, 3142, 2143, 3412, 2413 \}. $$

To view as signed permutations, it is helpful to define the operation $\overline{i}:= 2n + 1 - i$ for $1 \le i \le n$ and think of $\overline{i}$ as the reversed orientation of *i*. Then the permutations $\sigma \in B_n \subset S_{2n}$ satisfy $\sigma (\overline{i}) = \overline{\sigma (i)}$. For example, when n=2, we have $\overline{2} = 3, \overline{1} = 4$, and so

$$ 3142 = \overline{2} 1 \overline{1} 2. $$

Since the first *n* symbols uniquely determine the element in $B_n$, when it is clear we will omit the second half, e.g. $3142 = \overline{2} 1$.

#### Remark 1

Every element $x \in B_n$ has a decomposition into disjoint *signed cycles*. A *positive* *r*-cycle is of the form $(a_1, \dots , a_r)\cdot (\overline{a_1}, \dots , \overline{a_r})$ (a permutation which does not reverse the orientation of any labels) and a *negative* *r*-cycle is of the form $(a_1, \dots , a_r,\overline{a_1}, \dots , \overline{a_r})$ (in addition to permuting, the orientation of each label is reversed). The *signed cycle type* of *x* is the number of positive and negative cycles of different lengths. For example, $x = \overline{1}32 = (1 \overline{1})(23)(\overline{2}\overline{3})$ has one negative 1-cycle and two positive 2-cycles. It is a useful fact that two elements of $B_n$ are conjugate in $B_n$ if and only if they have the same *signed cycle type* (James and Kerber 1981).

#### Remark 2

More generally, $C_2$ can be replaced by any cyclic group $C_r$, r≥2, yielding the wreath product $C_r \wr S_n \cong C_r^n \rtimes S_n$, which would model genomes in which each gene has *r* possible orientations. Values r>2 do not have a clear biological interpretation but may be mathematically interesting.

***Dihedral subgroup*** To consider a circular genome with oriented regions, we will need to account for possible symmetries. The dihedral group $D_n \subset S_n$ is the group of symmetries of a regular polygon with *n* sides. It is generated by two elements: *r* (rotation) and *f* (reflection), and the relations $r^n = f^2 = (f \cdot r)^2 = id$. The order is $|D_n| = 2n$. We can extend $D_n$ to be embedded in $B_n$ as a subgroup of $S_{2n}$, generated by the rotation,

$$ r = (123 \ldots n) \cdot (\overline{1} \ldots \overline{n}) $$

and the reflection

1 $$\begin{aligned} f = \prod _{i = 1}^n (i, \overline{n + 2 - i}), \end{aligned}$$

where all addition is taken $\bmod \,n$ with the convention 0≡n (so that labels remain in $\{1, \ldots , n\}$ rather than $\{0, 1, \dots , n-1\}$). The reflection could be defined across any axis of the polygon with sides labeled 1,2,⋯,n; this choice corresponds to the axis through the side labeled 1.

#### Example 1

When n=3, the group $D_n \subset B_n$ is generated by

$$\begin{aligned}&r = (123)(654) = (123)(\overline{1}\overline{2}\overline{3}) = 231 \\&f = (24)(53)(16) = (2 \overline{3})(\overline{2} 3)(1 \overline{1}) = \overline{1} \overline{3} \overline{2} \end{aligned}$$

**Fig. 1:**
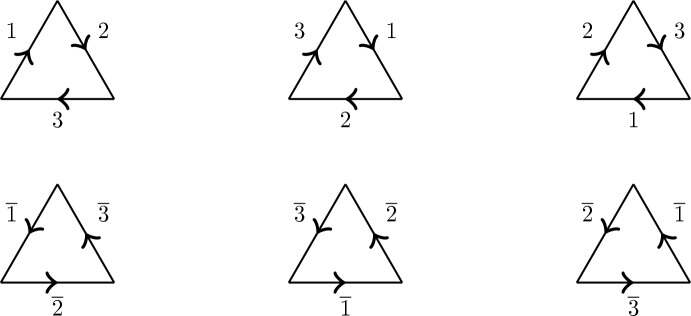
An example visualization of the dihedral group $D_3 \subset B_3$. Arrows on each edge indicate gene orientation: an arrow pointing clockwise indicates positive orientation, while an arrow pointing counterclockwise indicates reversed orientation

The following elements are depicted in Fig. 1:

$$\begin{aligned}&123, 312, 231 \\&\overline{1}\overline{3} \overline{2}, \overline{3} \overline{2} \overline{1}, \overline{2} \overline{1} \overline{3} \end{aligned}$$

***Young subgroup*** Finally, to account for genomes with repeated unique genes/regions, let $\lambda = (\lambda _1, \dots , \lambda _k)$ be a partition of *n*. That is, $\lambda _1 \ge \lambda _2 \ge \ldots \ge \lambda _k > 0$ are integers with $\sum _i \lambda _i = n$. The parts of λ determine the counts of genes which are indistinguishable, i.e. $\lambda _i$ genes of type ‘*i*’. For example, $\lambda = (1, 1, \dots , 1) = 1^n$ indicates all *n* genes are unique; λ=(2,2,1) indicates there are two genes of type ‘1’, two genes of types ‘2’, and one gene of type ‘3’. The labels are assigned in order of frequency so that type ‘1’ is the most commonly occurring gene, type ‘2’ is the second most common, and so on. Let $S_\lambda \subset S_n$ be the Young subgroup determined by λ. That is, $S_\lambda \cong S_{\lambda _1} \times S_{\lambda _2} \times \ldots \times S_{\lambda _k}$ is the set of all permutations with only the labels $1, \dots , \lambda _1$ permuted amongst themselves, only $\lambda _1 +1, \dots , \lambda _1 + \lambda _2$ permuted amongst themselves, and so on.

To embed $S_\lambda$ in $B_n$ as a subgroup of $S_{2n}$, extend each $\sigma \in S_\lambda$ by defining $\sigma (\overline{i}) = \overline{\sigma (i)}$ (or $\sigma (2n + 1 - i) = (2n + 1) - \sigma (i)$ for $1 \le i \le n$). This means that the size of the extended subgroup is still the same $|S_\lambda | = \lambda _1! \cdot \ldots \cdot \lambda _k!$.

#### Example 2

Suppose n=5 and λ=(2,2,1). Then the elements of $S_\lambda \subset B_n$, are

$$\begin{aligned}&12345 \rightarrow 12345 \overline{5} \overline{4} \overline{3} \overline{2} \overline{1} \\&21345 \rightarrow 21345 \overline{5} \overline{4} \overline{3} \overline{1} \overline{2} \\&12435 \rightarrow 12435 \overline{53421} \\&21435 \rightarrow 21435 \overline{53412} \\ \end{aligned}$$

These permutations differ only by swapping labels within the same gene type, i.e. they lie in the same $S_\lambda$ coset. As a reminder, elements of $B_n$ are written as words $\sigma (1)\cdots \sigma (n)$ of length *n* and the values on $\{\bar{1},\ldots ,\bar{n}\}$ are then determined by $\sigma (\bar{i}) = \overline{\sigma (i)}$.

### Induced distribution

Based on the discussion in the previous section, the double cosets $S_\lambda \backslash B_n / D_n$ are equivalence classes of $B_n$ with $D_n$ symmetry between the positions and $S_\lambda$ symmetry between the labels.

#### Example 3

For n=3,λ=(2,1) there are 4 double cosets $S_\lambda \backslash B_n / D_n$ each with 12 elements:

1. $$S_\lambda \backslash id / D_n = \left\{ 123, 213, 312, 321, 231, 132, \overline{1}\overline{3} \overline{2}, \overline{2 3 1}, \overline{3 2 1}, \overline{3 1 2}, \overline{2 1 3}, \overline{1 2 3} \right\} $$
2. $$S_\lambda \backslash 12 \overline{3} / D_n = \left\{ 12 \overline{3}, 21 \overline{3}, \overline{3} 12, \overline{3} 21, 2 \overline{3} 1, 1 \overline{3} 2, 3 \overline{2} \overline{1}, 3 \overline{1} \overline{2}, \overline{21} 3, \overline{12} 3, \overline{1}3\overline{2}, \overline{2}3 \overline{1} \right\} $$
3. $$S_\lambda \backslash \overline{1} 23 / D_n = \left\{ \overline{1}23, \overline{2}13, 23\overline{1}, 13\overline{2}, 3\overline{1}2, 3\overline{2}1, \overline{3}\overline{2}1, \overline{3}\overline{1}2, \overline{2}1\overline{3}, \overline{1}2\overline{3}, 1\overline{32}, 2\overline{31} \right\} $$
4. $$S_\lambda \backslash 1\overline{2} 3 / D_n = \left\{ 1\overline{2}3, 2\overline{1}3, \overline{2}31, \overline{1}32, 31\overline{2}, 32\overline{1}, \overline{3}2\overline{1}, \overline{3}1\overline{2}, 2\overline{1}3, 1\overline{2}3, \overline{1}32, \overline{2}31 \right\} $$

**Fig. 2:**
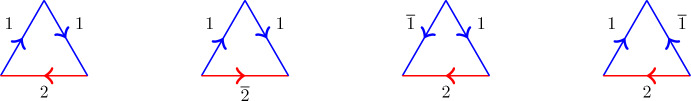
A visualization of the double cosets $S_{(2, 1)} \backslash B_3 / D_3$. From left to right, these correspond to double cosets (1), (2), (3), and (4) listed in Example 3

These 4 double cosets correspond to the unique circular genomes with two unique regions, one region which is repeated twice, as depicted in Fig. 2.

In Example 3 the double cosets are all the same size, which is $|S_{(2, 1)}|\cdot |D_3|$. In general, if *H*, *K* are subgroups of a finite group *G*, then the size of the double coset containing x∈G is

2 $$\begin{aligned} |HxK| = \frac{|H| \cdot |K|}{|K \cap x^{-1} H x|} = \frac{|H| \cdot |K|}{| x K x^{-1} \cap H|} = \frac{|H| \cdot |K|}{ |xK \cap Hx|} \end{aligned}$$

The denominator |xK∩Hx| is the intersection of the left coset *G*/*K* which contains *x* with the right coset H\G which contains *x*. For example, the size of the double coset containing the identity is simply:

$$ |H id K| = \frac{|H| |K|}{|H \cap K|}. $$

#### Remark 3

Equation (2) is an instance of the orbit-stabilizer theorem for the group H×K acting on *G* by $(h, k) \cdot g = hgk^{-1}$. The double coset *HxK* is precisely the orbit of *x* under this action and the stabilizer of *x* is $\{(h,k) \in H \times K: hxk^{-1} = x\} \cong K \cap x^{-1}Hx$, which has size $|K \cap x^{-1}Hx|$. The related Burnside’s Counting Lemma gives the total number of double cosets from the same action: $|H \backslash G / K| = \frac{1}{|H||K|} \sum _{(h,k) \in H \times K} |\{g \in G: hgk^{-1} = g\}|$. In the special case $G = S_n$, $K = D_n$, $H = S_\lambda$, this recovers the classical formula for counting necklaces with $\lambda _i$ beads of color *i* up to rotation and reflection.

Note that for all $\lambda \ne (n)$, Eq. (2) gives $|S_\lambda id D_n| = |S_\lambda | \cdot |D_n|$ because $D_n \cap S_\lambda = \{id \}$. In Example 3, it was also true that $xD_3 \cap S_\lambda x = \{id \}$ for all elements $x \in B_n$ and so the double cosets are all the same size. This is not always the case for more complicated λ. To count the size of $x D_n \cap S_\lambda x$, we introduce the concept of ‘coloring’ or re-labeling a permutation according to the partition λ.

#### Definition 1

Let $\lambda = (\lambda _1, \dots , \lambda _k)$ be a partition of *n*. For a permutation $x \in S_n$, define $x_\lambda$ as the coloring of *x* according to λ. The labels $1, \dots , \lambda _1$ are relabeled 1, labels $\lambda _1 + 1, \dots , \lambda _1 + \lambda _2$ are relabeled 2, and so on. Thus $x_\lambda$ is a word with letters 1,⋯,k and letter *j* occurs exactly $\lambda _j$ times. Similarly, for $x \in B_n$ the coloring *x* according to λ is a word with letters $1, \dots , k, \overline{1}, \dots , \overline{k}$. We continue to write elements of $B_n$ as words x(1)x(2)⋯x(n) of length *n* with symbols from $\{1, \dots , n, \overline{1}, \dots , \overline{n}\}$, so $x_\lambda$ is likewise a word of length *n*.

Two elements have the same coloring according to λ if they are in the same right coset of $S_\lambda$. That is, $x_\lambda = y_\lambda$ if and only if $x_\lambda \in S_\lambda y$.

#### Definition 2

For $x \in B_n$, the *rotational period* according to a partition λ is the minimum number of rotations before the coloring $x_\lambda$ repeats itself. That is,

$$ p_\lambda (x):= \min \{m> 0: x_\lambda \cdot r^m = x_\lambda \} = \min \{m >0: x\cdot r^m \in S_\lambda x \}. $$

#### Example 4

To illustrate this concept, consider n=4 and λ=(2,2).

1. The element $x = 1234 \in B_n$ relabeled according to λ is 1122 with rotational period $p_\lambda (x) = 4$.
2. The element $x = 1324 \in B_n$ relabeled according to λ is 1212 with rotational period $p_\lambda (x) = 2$.

The size of a double coset also depends on whether any rotation of $x_\lambda$ is invariant under the reflection *f* (defined in (1)). If $x_\lambda \cdot f = x_\lambda$ for some rotation of $x_\lambda$, the double coset is smaller because the reflection does not produce a new element. For example, the coloring $x_\lambda = 1\overline{2}\,2\overline{1}$ satisfies $x_\lambda \cdot f = x_\lambda$: reversing the sequence and flipping orientations gives $1\overline{2}\,2\overline{1}$ again.

#### Definition 3

Suppose *n* is even and λ is a partition with all even parts. Define $x \in B_n$ to be *anti-palindromic* with respect to λ if $x_\lambda \cdot f = x_\lambda$. Equivalently,

$$ x(1), \dots , x(n/2) = \overline{x(n), \dots , x(n/2 + 1)}. $$

That is, the second half of the word *x* is the reflection of the first half.

#### Theorem 1

For a partition λ of *n*, define

$$\begin{aligned} c_\lambda (x) := {\left\{ \begin{array}{ll} 1 & \text {if} \,\,\, x_\lambda \cdot r^\ell \,\,\, \text {is anti-palindromic for some} \,\,\, \ell \\ 2 & \text {otherwise} \end{array}\right. }. \end{aligned}$$

The size of the double coset $S_\lambda \backslash B_n / D_n$ containing $x \in B_n$ is:

$$\begin{aligned} |S_\lambda x D_n| = |S_\lambda | \cdot p_\lambda (x) \cdot c_\lambda (x). \end{aligned}$$

#### Proof

We will prove the result by showing $|S_\lambda x \cap x D_n| = 2n/(p_\lambda (x) \cdot c_\lambda (x))$. Suppose $p_\lambda (x) = p$. Then $x\cdot r^{p \cdot k} \in S_\lambda x$ for all *k* and $x \cdot r^{p \cdot k}$ are unique elements in $B_n$ for k=0,1,⋯n/p-1 (since *r* has order *n*, these are distinct for *k* in this range). If there are no rotations which make $x_\lambda$ anti-palindromic ( and so $c_\lambda (x)=2$), then $x \cdot r^\ell f \notin S_\lambda x$ for any ℓ and exactly $|S_\lambda x \cap x D_n| = n/p_\lambda (x)$.

If $x_\lambda r^\ell$ is anti-palindromic for some ℓ then $x_\lambda r^\ell f = x_\lambda r^\ell$. This also gives

$$ x_\lambda \cdot r^\ell f \cdot r^{p\cdot k - \ell } = x_\lambda \cdot r^{p \cdot k} = x_\lambda , \quad k = 0, 1, \dots , n/p - 1. $$

Thus, these are *n*/*p* additional unique elements in $B_n$ which are in the intersection $S_\lambda x \cap x D_n$ (they are distinct among themselves since $r^{pk}$ are distinct, and distinct from the rotation elements since $f \ne r^m$ for any *m*), and so in this case $|S_\lambda x \cap x D_n| = 2 n/p_\lambda (x)$. Formula 2 shows

$$\begin{aligned} |S_\lambda x D_n| =\frac{|S_\lambda | \cdot |D_n|}{2n/(p_\lambda (x) \cdot c_\lambda (x))} = |S_\lambda | \cdot p_\lambda (x) \cdot c_\lambda (x). \end{aligned}$$

□

#### Corollary 2

The probability distribution on double cosets $S_\lambda \backslash B_n / D_n$ induced by the uniform distribution on $B_n$ is:

$$ \pi (S_\lambda x D_n):= \frac{\lambda _1 ! \ldots \lambda _k ! \cdot p_\lambda (x) \cdot c_\lambda (x)}{2^{n} \cdot n! }, \quad x \in B_n. $$

#### Example 5

Consider n=6,λ=(4,2). Writing $\pi (S_\lambda x D_n) = \pi (x_\lambda )$, example probabilities for different double cosets are:

$$\begin{aligned}&\pi (112112) = \frac{4! \cdot 2! \cdot 3 \cdot 2}{2^6 \cdot 6!} = \frac{1}{160} \\&\pi (112 \overline{211}) = \frac{4! \cdot 2! \cdot 6 \cdot 1}{2^6 \cdot 6!} = \frac{1}{160}\\&\pi (1121\overline{2}1) = \frac{4! \cdot 2! \cdot 6 \cdot 2}{2^6 \cdot 6!} = \frac{1}{80} \end{aligned}$$

The first example has $p_\lambda (x) = 3, c_\lambda (x) = 2$, the second has $p_\lambda (x) = 6, c_\lambda (x) = 1$, and the third has $p_\lambda (x) = 6, c_\lambda (x) = 2$.

The uniform distribution on $B_n$ is the natural choice in the absence of any prior biological information, treating all initial gene orderings and orientations as equally likely before accounting for symmetries. This provides a reference distribution against which the output of any specific rearrangement process can be compared, and serves as the stationary distribution of the double coset Markov chains introduced in Sect. 3.

### Counting double cosets

The formula in Theorem 1 shows that the possible sizes of the double cosets, and thus the induced probability distribution on genomes, depends greatly on the structure of the partition $\lambda = (\lambda _1, \lambda _2, \dots , \lambda _k)$. A few simple observations on possibilities for $p_\lambda (x)$ and $c_\lambda (x)$:

- If $\lambda _i = 1$ for some *i*, then necessarily $p_\lambda (x) = n$.
- If *x* has rotational period $p_\lambda (x) = p$, then necessarily p∣n. If p=n/s for some integer $1 \le s \le n$, then either label *i* or $\overline{i}$ must occur at least *s* times. That is $\lambda _i \ge s$ for all *i* and so $n/\min (\lambda _i) \le p \le n$.
- If the rotational period is *p*, there are s=n/p repeated blocks in the sequence. Since label *i* or $\overline{i}$ occurs the same number of times in each block and total $\lambda _i$, it must be that $n/p \mid \lambda _i$ for all *i*.
- If $\lambda _i$ is odd for some *i*, then there are no anti-palindromic sequences ($c_\lambda (x) = 2$ for all *x*).

Using these properties, we can compute the number of double cosets in special cases. The general formula for $|S_\lambda \backslash B_n / D_n|$ for arbitrary λ remains open; see Open Problem 1 in Sect. 4.

#### Proposition 3

If $\gcd (\lambda _1, \lambda _2, \ldots , \lambda _k) = 1$, then every double coset has the same size $2n \cdot |S_\lambda |$ and the number of double cosets is

$$ |S_\lambda \backslash B_n / D_n| = \frac{2^n \cdot n!}{2n \cdot |S_\lambda |} = \frac{2^{n-1} \cdot (n-1)!}{\lambda _1! \ldots \lambda _k!}. $$

#### Proof

We show $p_\lambda (x) = n$ and $c_\lambda (x) = 2$ for all $x \in B_n$, so that Theorem 1 gives $|S_\lambda x D_n| = 2n\cdot |S_\lambda |$ uniformly. If $p_\lambda (x) = p$, then the coloring $x_\lambda$ consists of *n*/*p* repeated blocks, and each label *i* or $\overline{i}$ appears the same number of times in each block. Since label *i* appears $\lambda _i$ times in total, we need $(n/p) \mid \lambda _i$ for every *i* and so $n/p \mid \gcd (\lambda _1, \dots , \lambda _k) = 1$. Hence n/p=1 and p=n. Finally, since $\gcd (\lambda _1, \dots , \lambda _k) = 1$, it must be $\lambda _i$ is odd for some *i*, so $c_\lambda (x) = 2$ for all *x*. □

Proposition 3 is likely the most common biological situation in long and complex genomes, especially the case $\lambda _i = 1$ for some *i*. Even in highly repetitive genomes there is typically at least one unique gene or genomic landmark (such as an origin of replication) that breaks full repetitive symmetry (Bader 2010; Sankoff 2001). In this case, since all double cosets are equal size, the uniform distribution on $B_n$ induces the uniform distribution on genome configurations. This is a natural null model of unbiased random rearrangement. In contrast, the following proposition is for the case $\lambda = \textbf{2}^m$, which results in a non-uniform induced distribution. This case is mathematically tractable but unlikely to arise in most biological settings. It is included as an illustrative example of the counting technique.

#### Proposition 4

If n=2m for some integer m≥2 and $\lambda = \textbf{2}^m = (2, 2, \dots , 2)$, then the number of double cosets is

$$ |S_\lambda \backslash B_n / D_n| = 2^{m-2} \cdot \left( 2\cdot (2\,m-1)! + m! + (m-1)! \right) . $$

#### Proof

We will consider the possible sizes of double cosets. Recall that if $x \in B_n$, the coloring $x_\lambda$ consists of the labels 1,2,⋯,m and $\overline{1}, \overline{2}, \dots , \overline{m}$, such that total occurrences of *i* and $\overline{i}$ is $\lambda _i = 2$. There are three possibilities based on $p_\lambda (x)$ and $c_\lambda (x)$:

1. $p_\lambda (x) = m$: This occurs for *x* such that $x_\lambda$ is the same sequence of length *m* repeated twice. There are $m! \cdot 2^m$ possible sequences. However, since rotating the sequence or reflecting will give sequences in the same double coset, there are $m! \cdot 2^m/(2m) = (m-1)! \cdot 2^{m-1}$ unique double cosets. The division by $2m = |D_{2m}|$ is valid because every element of $D_{2m}$ acts freely on these sequences. No non-identity rotation or reflection fixes an aperiodic sequence of this type, so all 2*m* dihedral images are distinct.
2. $p_\lambda (x) = 2m, c_\lambda (x) = 1$: These are the anti-palindromic sequences (which must necessarily be aperiodic with respect to λ). To specify an anti-palindromic sequence, one can define the first *m* elements (with *i* or $\overline{i}$ occurring exactly once) and set the second *m* elements as the reflection of the first. There are initially $m! \cdot 2^m$ possibilities, but since reflections remain in the double coset, there are $m! \cdot 2^{m-1}$ unique double cosets. This is because no non-trivial rotation fixes $x_\lambda$, while exactly one reflection does (by definition of anti-palindromicity, $c_\lambda (x)=1$). Thus each double coset contains exactly 2 colorings.
3. $p_\lambda (x) = 2m, c_\lambda (x) = 2$: These are the sequences that are not anti-palindromic and are aperiodic with respect to λ. Suppose the number of unique double cosets in this setting is *A*.

To determine *A*, we use the formula for the sizes of double cosets from Theorem 1 (see Table 1) and the total size of $|B_n|$. This gives the equation

$$\begin{aligned} |B_n|&= (2m)!\cdot 2^{2m} = A \cdot (m\cdot 2^{m+2}) + (m!\cdot 2^{m-1})\cdot (m\cdot 2^{m+1}) \\&\quad + \left( (m-1)!\cdot 2^{m-1} \right) \cdot (m\cdot 2^{m+1}). \end{aligned}$$

Solving this equation gives

$$ A = 2^{m-2}\cdot \left( 2(2\,m-1)! - (m+1)\cdot (m-1)! \right) . $$

Now using this expression, the total number of double cosets is the sum of the three counts in Table 1:

$$\begin{aligned} |S_\lambda \backslash B_n / D_n|&= (m-1)!\cdot 2^{m-1} + m!\cdot 2^{m-1} + 2^{m-2}\bigl (2(2m-1)! - (m+1)(m-1)! \\&= 2^{m-2}\left( 2(m-1)! + 2\cdot m! + 2(2m-1)! - (m+1)(m-1)! \right) \\&= 2^{m-2}\left( 2(2m-1)! + 2\cdot m! + (1-m)(m-1)!\right) . \end{aligned}$$

Since 2·m!+(1-m)(m-1)!=(m+1)(m-1)!=m!+(m-1)!, this simplifies to

$$ |S_\lambda \backslash B_n / D_n| = 2^{m-2}\bigl (2(2\,m-1)! + m! + (m-1)!\bigr ). $$

**Table 1:** The three possibilities for double cosets with $\lambda = \textbf{2}^m$

Example $x \in B_n$	Example $x_\lambda \in S_\lambda /B_n$	$p_\lambda (x)$	$c_\lambda (x)$	$|S_\lambda x D_n|$	Number of DC of this type
135246	123123	*m*	2	$m\cdot 2^{m+1}$	$(m-1)!\cdot 2^{m-1}$
$123\overline{654}$	$123\overline{321}$	2*m*	1	$m\cdot 2^{m+1}$	$m!\cdot 2^{m-1}$
$1\overline{3}54\overline{6}2$	$1\overline{2}32\overline{3}1$	2*m*	2	$m\cdot 2^{m+2}$	*A*

The first two columns are examples of specific elements for m=3, n=6. The fifth column is $|S_\lambda x D_n| = 2^m \cdot p_\lambda (x) \cdot c_\lambda (x)$

□

## Markov chains

**Fig. 3:**
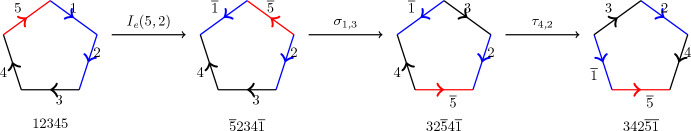
Example of different rearrangement moves on a genome with n=5 and λ=(2,2,1). Below each pentagon are representative elements from $B_n$. The first move $I_e(5, 2)$ inverts the segment formed by regions 5 and 1. The second move $\sigma _{1,3}$ transposes the regions at positions 1 and 3. The third move $\tau _{4, 2}$ translocates the region at position 4 to position 2

In this section, we discuss how to define several possible Markov chains on the space of double cosets $S_\lambda \backslash B_n /D_n$ which have interpretations as biological mutations of a circular genome with repeated segments. Section 3.1 reviews the theory of double coset Markov chains. Sections 3.2, 3.3, and 3.4 review the specific processes described by inversions, transpositions, and translocations, respectively. For each process, we note results for similar Markov chains on $S_n$ or $B_n$. Figure 3 shows an example sequence of transitions (notation described in the following sections).

### Double coset Markov chains

Let *H*, *K* be subgroups of a finite group *G*, and let *Q* be a probability on *G* (i.e. Q(s)≥0 for all s∈G and $\sum _{s \in G} Q(s) = 1$). If $\text {supp}(Q)$ is not contained in a coset of a subgroup, then the random walk on *G* induced by *Q* is ergodic with a uniform stationary distribution (Diaconis 1988). This random walk is defined by picking a random element from *G* with probabilities given by *Q* and multiplying the current state on either the left or right. That is, transition probabilities are

$$ P(x, y) = Q(yx^{-1}) \quad \quad \text {or} \quad \quad P(x, y) = Q(x^{-1}y), \quad \quad x, y \in G. $$

See Diaconis (1988) for an introduction to random walks on groups.

The double cosets of H\G/K partition the space *G* and any Markov chain on *G* defines a random process on the set of double cosets by keeping track of which double coset the process on *G* is in at each step. This induced process on equivalence classes is called the *lumped process*. Proposition 5 gives a condition for when the random walk induced by *Q* on *G* is also a Markov chain on the double cosets. For the statement, we fix double coset representatives and write *x* for the double coset *HxK*. The result is proven in Diaconis et al. (2023) using Dynkin’s criterion. See Simper (2022) and Simper (2023) for more results and discussion.

#### Proposition 5

Let *Q* be a probability on *G*. If *Q* is *H*-conjugacy invariant ($$Q(s) = Q(h^{-1}s h) $$ for $$h \in H, s \in G$$), then multiplication on the left according to *Q* maps to a Markov chain on H\G/K with transition kernel

$$ P(x, y) = Q \left( HyKx^{-1} \right) = \sum _{h \in H} \sum _{k \in K} Q(hykx^{-1}). $$

If *Q* is *K*-conjugacy invariant, then multiplication on the right according to *Q* maps to a Markov chain on H\G/K with transition kernel

$$ P(x, y) = Q(x^{-1}HyK). $$

The stationary distribution is π(x)=|HxK|/|G|. If $Q(s) = Q(s^{-1})$, then (P,π) is reversible.

This result will be used in following sections by showing certain subsets of $B_n$ are $D_n$-conjugacy invariant and so a measure which assigns uniform probability to the subset will induce a Markov chain on double cosets. The similar necessary and sufficient condition that Q(kxHyK)=Q(xHyK) for all x,y∈G and k∈K (for a process defined by multiplication on the right) was noted in Britnell and Wildon (2024).

***Mixing times*** A classic object of study for Markov chains is the *mixing time* – the rate of convergence to stationarity. That is, for a Markov chain on a space Ω with transition probability *P* and stationary distribution π, the mixing time is

$$ t_{mix}(\epsilon ) = \sup _{x_0 \in \Omega } \inf \left\{ t> 0: \Vert P^t(x_0, \cdot ) - \pi (\cdot ) \Vert _{TV} < \epsilon \right\} , \quad \epsilon > 0, $$

where $\Vert \mu - \pi \Vert _{TV} = \sup _{A \subset \Omega } |\mu (A) - \pi (A)|$ is the total variation distance between probability measures. Most works use the standard $t_{mix}:= t_{mix}(1/4)$. The convergence rate is closely related to the eigenvalues and eigenfunctions of the Markov chain, which give an exact formula for the *chi-squared* distance between the chain and the stationary distribution. If $\beta _i$ denote the eigenvalues and $f_i(x)$ orthonormal eigenfunctions, then the chi-squared distance $\chi ^2_x(t)$ is related to the total variation distance and defined:

3 $$\begin{aligned} \Vert P^t(x, \cdot ) - \pi (\cdot ) \Vert _{TV}^2 \le \frac{1}{4}\chi ^2_x(t) := \frac{1}{4}\cdot \sum _{i=1}^{|\Omega | - 1} \beta _i^{2t} \cdot f_i^2(x). \end{aligned}$$

The sum runs over all |Ω|-1 non-trivial eigenvalues counted with multiplicity, so degenerate eigenspaces (eigenvalues with multiplicity >1) contribute one term per eigenfunction (see Chapter 12 of (Wilmer et al. 2009)). This formulation is useful for comparing the mixing time of a lumped process to the mixing time of the original chain, since the eigenvalues of the lumped process are some subset of the eigenvalues of the base chain. That is to say, the mixing time of the lumped process is no slower than the mixing time of the base chain, and it could be a great deal faster.

If the *Q* driving the Markov chain is more specifically a class function (constant on conjugacy classes of *G*), then the eigenvalues of the chain have explicit expressions in terms of the characters of irreducible representations of *G*. Furthermore, the multiplicities of the eigenvalues for the lumped chain H\G/K can be calculated using the number of times the trivial representation appear in the restrictions of the representations of *G* to *H* and *K* (see Theorem 1.2 in (Diaconis et al. 2023) for the precise statement and formulas). The irreducible representations of $B_n$ have been long known (e.g. Appendix B of Chapter I in (Macdonald 1998)) and are indexed by bi-partitions of *n* (pairs $\overline{\mu } = (\mu ^1, \mu ^2)$ of partitions with $|\mu ^1| + |\mu ^2| = n$).

### Inversions

An inversion in $B_n$ is a transposition $(i \overline{i}) = (i (2n + 1 - i))$ for $1 \le i \le n$. From the circular genome perspective, multiplication on the left by the inversion $(i \overline{i})$ reverses the orientation of label *i*. Multiplication on the right reverses the orientation of the gene at position *i*. More explicitly, we can define inversions of multiple consecutive genes/regions. Throughout, all addition is $\bmod \, n$.

#### Definition 4

An inversion of odd length 2m+1 centered at position *r* is

$$\begin{aligned} I_o(r, 2m+1) := (r, \overline{r})\prod _{i = 1}^m (r - i, \overline{r + i}) \cdot (\overline{r-i}, r +i) \end{aligned}$$

An inversion of even length 2*m* about the pair of regions r,r+1 is

$$\begin{aligned} I_e(r, 2m) = \prod _{i = 1}^m (r - (i-1), \overline{r + i})\cdot (\overline{r - (i - 1)}, r + i). \end{aligned}$$

Let $\mathcal {I}_n(m)$ denote the set of all inversions of length *m* and $\mathcal {I}_n = \cup _{m = 1}^n \mathcal {I}_n(m)$ the set of all inversions of any length.

#### Remark 4

In $S_n$, the analogy of an inversion of length 2 is simply an adjacent transposition. The set of adjacent transpositions generates $S_n$. Similarly, the set of all inversions of length 1 or 2 generates $B_n$.

#### Lemma 6

Inversions of length one, $\mathcal {I}_n(1)$, form a conjugacy class in $B_n$. For any *m*, the set of all inversions of length *m*, $\mathcal {I}_n(m)$, is $D_n$-conjugacy invariant in $B_n$.

#### Proof

It suffices to prove that $\mathcal {I}_n(1)$ is conjugacy invariant by generators of $B_n$. We can take $B_n$ to be generated by $\mathcal {I}_n(1) \cup \mathcal {I}_n(2)$. If $h, x \in \mathcal {I}_n(1)$, then

$$\begin{aligned} xhx^{-1} = x^{-1}hx = h. \end{aligned}$$

If $h = (i, \overline{i}) \in \mathcal {I}_n(1)$ and $x = (j, \overline{j+1}), (j+1, \overline{j}) \in \mathcal {I}_n(2)$, then

$$\begin{aligned} xhx^{-1} = x^{-1}hx = {\left\{ \begin{array}{ll} h & \text {if} \,\, j, j+1 \ne i \\ (i+1, \overline{i+1}) & \text {if} \,\, j = i \\ (i-1, \overline{i-1}) & \text {if} \,\, j+1 = i. \end{array}\right. } \end{aligned}$$

Now to show $\mathcal {I}_n$ is $D_n$-conjugacy invariant, we will check conjugacy invariance under the generators *f*, *r* of $D_n$. For completeness and to record formulas, we will check both left and right conjugacy invariance, though the proof of only one is necessary. Note that for the reflection $f = f^{-1}$ and for the rotation

$$ r = (123 \dots n) \implies r^{-1} = (n(n-1) \dots 321). $$

We compute:

$$\begin{aligned} fI(a, m)f = {\left\{ \begin{array}{ll} I(n + 2 - a, m) & \text {if } \,\, m \,\, \text {odd} \\ I(n + 1 - a, m) & \text {if } \,\, m \,\, \text {even} \end{array}\right. }. \end{aligned}$$

and

$$\begin{aligned}&rI(a, m)r^{-1} = I(a+1, m) \\&r^{-1}I(a, m) r = I(a-1, m). \end{aligned}$$

□

#### Remark 5

From Remark 1, an inversion of odd length 2m+1 has signed cycle type consisting of one negative 1-cycle and *m* positive 2-cycles and an inversion of even length 2*m* has *m* positive 2-cycles. That is, all inversions of the same length share the same signed cycle type. The first part of Lemma 6 follows directly since $\mathcal {I}_n(1)$ is exactly the set of all possible negative 1-cycles, and is thus a conjugacy class. However, for m>1, $\mathcal {I}_n(m)$ is not a conjugacy class because there are elements of the same signed cycle type which are not inversions. For instance, $(1 \overline{3})(3 \overline{1})$ is a negative 2-cycle and has the same signed cycle type as elements of $\mathcal {I}_n(2)$, but is not an inversion.

Proposition 5 says any probability measure that gives equal weight to inversions of the same length induces a Markov chain on $S_\lambda \backslash B_n / D_n$ by multiplication on the right.

#### Example 6

*(Inversion Markov Chain)* Suppose $\{\alpha _j \}_{j = 1}^n$ is a set with $0 \le \alpha _j \le 1$ and $\sum _j \alpha _j = 1$. Define a probability measure *Q* on $B_n$ such that the probability of any inversion of length *j* is $\alpha _j$ and each inversion of length *j* is equally likely. That is, each individual inversion has probability $\alpha _j/n$, since there are are *n* inversions of length *j*, and so

$$\begin{aligned} Q(\omega ) = \frac{1}{n} \sum _{j = 1}^n \alpha _j \cdot \textbf{1}\left( \omega \in \mathcal {I}_n(j) \right) . \end{aligned}$$

Then *Q* induces a Markov chain on double cosets $S_\lambda \backslash B_n / D_n$ by multiplication on the right. This setting allows for flexibility in defining the relative probabilities of inversions of different length. For example, an analysis of *Drosophila* species mutatations noted very small inversions are rare and evolutionarily successful mutations are intermediate in length (Cáceres et al. 1997).

Inversion Markov chains are similar to the so-called *L*-reversal chain introduced and studied in (Durrett 2008, 2003). This model on $S_n$ is defined for a fixed $1 \le L \le n$: Pick an integer $1 \le \ell \le L$ uniformly and pick an index $1 \le x \le n$ uniformly randomly, then reverse the segment at positions $x, x+1, \dots , x + \ell$ (with addition $\bmod \, n$). Durrett conjectured that the mixing time of this chain is $O(\max (n, n^3/L^3) \log n)$. The spectral gap was studied in (Cancrini et al. 2006) and the best-known upper bound of $O(\max (n, n^3/L^3) \log ^3 n)$ was proven in (Morris 2009). However, a full spectral analysis remains elusive.

### Transpositions

Transpositions are a classic move in the world of Markov chains on $S_n$. The random transpositions Markov chain was one of the first for which the mixing time was explicitly analyzed (Diaconis 1988). From the circular genome perspective, the effect of a transposition is to interchange two gene segments. This may not be as biologically plausible because it may require cuts at 4 locations in the genome. Note that two *adjacent* genes on a circular genome can be interchanged with a single cut and rejoin via site-specific recombination (Klippel et al. 1993; Francis 2014), making adjacent (signed) transpositions more biologically realistic.

#### Definition 5

A transposition in $B_n$ is a signed permutation

$$ \sigma _{i,j}:= (i,j)(\overline{i},\overline{j}), \quad \quad 1 \le i \ne j \le n. $$

#### Lemma 7

Transpositions are $D_n$-conjugacy invariant in $B_n$.

#### Proof

We will check that transpositions are left and right conjugacy invariant under the generators *f*, *r* of $D_n$. That is,

$$\begin{aligned}&r^{-1} \sigma _{i,j} r = (i+1,j+1)(\overline{i+1}, \overline{j+1}) = \sigma _{i+1,j+1} \\&r \sigma _{i,j} r^{-1} = (i-1,j-1)(\overline{i-1}, \overline{j-1}) = \sigma _{i-1,j-1} \\&f\sigma _{i,j} f = \sigma _{n+2-i, n+2 - j}. \end{aligned}$$

□

#### Remark 6

The proof of Lemma 7 also shows that *adjacent transpositions* are $D_n$-conjugacy invariant in $B_n$. Thus a Markov chain which only transposed adjacent segments of a genome with equal probability would be well-defined. (Similar to an inversion of length 2, except the original orientations are preserved.)

#### Remark 7

In addition, the notion of transpositions could be extended to *block-interchange* rearrangement events where a sequence of more than one segment is transposed with another sequence of more than one segment (Stevenson et al. 2023). If the two sequences are the same length, then the block-interchange move is equivalent to applying a sequence of disjoint transpositions. The proof of Lemma 7 adapts to show the set of all block-interchange rearrangements of a fixed length are $D_n$-conjugacy invariant.

Note that transpositions which only preserve the orientation of the transposed genes would define a Markov chain which is not connected. To account for this, a connected Markov chain must also have some probability of reversing the orientation of gene segments. So-called ‘flip-transpose’ Markov chains on $B_n$ have been defined in various ways e.g., (Schoolfield, 2005; Matheau-Raven, 2020), having independent probability 1/2 of reversing the orientation of each of the transposed segments. We can define the version from Matheau-Raven (2020) using the notation of inversions of length 1 from the previous section:

#### Example 7

*(Flip-Transpose Markov Chain)* Consider the probability measure on $B_n$ defined

$$\begin{aligned} Q(\omega ) = {\left\{ \begin{array}{ll} 1/2n & \text {if} \quad \omega = id \\ 1/2n^2 & \text {if} \quad \omega = I_o(i, 1) \quad i \in [n] \\ 1/n^2 & \text {if} \quad \omega = \sigma _{i, j} \quad i \ne j \in [n] \\ 1/n^2 & \text {if} \quad \omega = I_o(i, 1)\cdot I_o(j, 1) \cdot \sigma _{i, j} \quad i \ne j \in [n] \\ 0 & \text {otherwise} \end{array}\right. }. \end{aligned}$$

Then *Q* induces a Markov chain on double cosets $S_\lambda \backslash B_n / D_n$ by multiplication on the right.

Because this measure is constant on conjugacy classes, some analysis is made easier and the full spectrum of this flip-transpose hyperoctahedral Markov chain was found in Matheau-Raven (2020) (Theorem 4.3.2) via a correspondence between the eigenvalues and standard Young bi-tableaux. This is used to show the mixing time is tightly bounded by (n/2)log(n). Since the chain on double cosets is a lumping of the process on $B_n$, this gives an immediate upper bound (Section 4.3.1 in (Matheau-Raven 2020)). In addition, because *Q* is a class function, Theorem 1.2 in Diaconis et al. (2023) applies to give an expression for the multiplicities of the eigenvalues using irreducible representations of $B_n$. The irreducible representations of $B_n$ are indexed by bi-partitions of *n* ($\overline{\mu } = (\mu ^1, \mu ^2)$, where $\mu ^1, \mu ^2$ are partitions of some integers $n_1, n_2$ with $n_1 + n_2 = n$). For the statement of the result, let $\chi _{\overline{\mu }}$ denote the representation, $\chi _{\overline{\mu }}|_H$ the restriction to a subgroup *H* of $B_n$ and $\left\langle \chi _{\overline{\mu }}|_H, 1 \right\rangle$ is the the number of times the trivial representation of *H* appears in $\chi _\lambda |_H$. The *Durfee size* of a partition is denoted $\textrm{Diag}(\lambda ) = \max \{i \ge 1: \mu _i \ge i \}$.

#### Theorem 8

(From (Matheau-Raven 2020), Theorem 4.3.2 and (Diaconis et al. 2023), Theorem 1.2.) The eigenvalues of the flip-transpose Markov chain on $S_\lambda \backslash B_n / D_n$ are indexed by bi-partitions $\overline{\mu } = (\mu ^1, \mu ^2)$ of *n*. The eigenvalue corresponding to $\overline{\mu }$ is defined

$$ \textrm{eig}(\overline{\mu }) = \frac{1}{2n^2} \left( 2 | \mu ^1| + 4 \cdot \textrm{Diag}(\mu ^1) + 4 \cdot \textrm{Diag}(\mu ^2) \right) . $$

The multiplicity is

$$\begin{aligned} m(\overline{\mu }) = \left\langle \chi _{\overline{\mu }}|_{S_\lambda }, 1 \right\rangle \cdot \left\langle \chi _{\overline{\mu }}|_{D_n}, 1 \right\rangle . \end{aligned}$$

Some observations from the formulas in Theorem 8:

1. The eigenvalue $\textrm{eig}(\overline{\mu })$ depends only on the bi-partition $\overline{\mu }$ and the ambient group $B_n$, not on the partition λ. Thus the *spectrum* of the flip-transpose chain is the same for all repeated-gene structures λ.
2. The multiplicity $m(\overline{\mu })$ depends on both λ (through the restriction $\chi _{\overline{\mu }}|_{S_\lambda }$) and *n* (through the restriction $\chi _{\overline{\mu }}|_{D_n}$). In particular, $\overline{\mu }$ contributes nontrivially to the lumped chain if and only if $\chi _{\overline{\mu }}$ contains the trivial representation when restricted to *both* $S_\lambda$ and $D_n$ simultaneously.
3. The spectral gap of the lumped chain is $1 - \max _{\overline{\mu }\,:\, m(\overline{\mu }) > 0,\, \overline{\mu } \ne \overline{(n)}} \textrm{eig}(\overline{\mu })$, where $\overline{(n)}$ denotes the trivial bi-partition. This gap controls the rate of convergence to stationarity via the chi-squared bound Eq. 3.

### Translocations

Translocations are a rearrangement move formed by cutting one region or segment of a genome and reinserting it at a different position. In this way it differs from a transposition because a maximum of 3 cuts are required. Often translocations are discussed in the context of multiple chromosomes. However, they can also be defined in the setting of a single chromosome, such as transposons within a bacterial genome. For our definition, we take the convention that a segment at position *j* is moved to position *m* by inserting it between positions *m* and +1. Thus, the segments at positions m,m-1,⋯,j+1 shift down by one and the segments at positions m+1,m+2,⋯,j-1 remain fixed. As before, all addition is computed $\bmod \, n$ (and thus the definition remains consistent regardless of j>m or j<m).

#### Definition 6

A *translocation* in $B_n$ which moves a region at position *j* to position *m* is the signed permutation

$$ \tau _{j, m} = (j, m, m-1, \dots , j+1) \cdot (\overline{j}, \overline{m}, \overline{m-1}, \dots , \overline{j+1}) $$

#### Lemma 9

The set of translocations is $D_n$-conjugacy invariant in $B_n$.

#### Proof

We prove that translocations are conjugacy invariant under the generators *f*, *r* of $D_n$ (again, checking both left and right conjugacy for completeness). First, if j<k, then

$$\begin{aligned}&r^{-1} \tau _{j, m} r = ( j+1, m+1, m, \dots , j+2) \cdot (\overline{j+1}, \overline{m+1}, \overline{m}, \dots , \overline{j+2}) = \tau _{j+1, m+1}\\&r \tau _{j, m} r^{-1} = (j-1, m-1, m-2, \dots , j) \cdot (\overline{j-1}, \overline{m-1}, \overline{m-2}, \dots , \overline{j}) = \tau _{j-1, m-1}. \end{aligned}$$

For conjugation by reflection:

$$\begin{aligned} f\tau _{j, m} f^{-1} = \tau _{n+2-j, n+2 - m}. \end{aligned}$$

□

This means that the set of translocations is closed under the dihedral symmetry of the genome: rotating or reflecting the genome before performing a translocation yields the same distribution on genome types as performing a translocation on the original genome. A translocation is most similar to a random-to-random or random-to-top shuffle on $S_n$, see e.g. Bernstein and Nestoridi (2019) for more definitions and background. For the hyperoctahedral group, a version of random-to-top shuffle with flips was studied in Ghosh (2021) and the mixing time was shown to be order nlogn.

## Discussion

This work presents a new framework for circular genomes with orientation and repeated genes as a double coset space. This perspective is useful for defining transitions to model genome rearrangement events, building on the double coset Markov chain framework developed in (Diaconis et al. 2023). The double coset setting expands on the Markov chains studied in Terauds and Sumner (2022); Stevenson et al. (2023) to allow not only circular symmetry, but also repeated genes. In addition, the double coset description shows how existing results on mixing times and eigenvalues for random walks on $B_n$ (such as those for the flip-transpose chain in Sect. 3.3) can be applied directly to bound the rate of convergence for processes on genomes. We note several open problems to explore.

***Open problem 1:*** What is the size of $S_\lambda \backslash B_n /D_n$ for a general λ?

The formulas in Sects. 2.2 and 2.3 show the sizes and number of double cosets greatly depends on the partition λ. Though the count can simplify significantly in certain cases (e.g. $\gcd (\lambda _1, \dots , \lambda _k) = 1$), a complete formula for the number of double cosets is elusive. Counting unlabeled or partially labeled objects is in general a difficult task and instead algorithms to approximately count may be needed, such as the Burnside process (Diaconis and Zhong 2026). In the sense of Diaconis et al. (2023), the ‘generic’ double coset is one of maximum size $|S_\lambda | \cdot 2n$, corresponding to $p_\lambda (x) = n$ and $c_\lambda (x) = 2$. By Proposition 3, this maximum size is achieved by every double coset when $\gcd (\lambda _1, \dots , \lambda _k) = 1$, which is also the biologically typical setting.

***Open problem 2:*** What are tight bounds for the mixing times of inversions, transpositions, and translocation Markov chains on $S_\lambda \backslash B_n /D_n$?

In general it is interesting to know if a lumped Markov chain mixes significantly faster than the original process. For example, the flip-transpose Markov chain in Sect. 3.3 mixes tightly in (n/2)log(n) steps on $B_n$. Is the lumped chain on double cosets the same order of magnitude? The representation theory of the hyperoctahedral group $B_n$ could be developed further to find the multiplicity of the eigenvalues in Theorem 8 for a lower bound. Similar analysis could be applied to the other Markov chains described in this paper and provide more examples in double coset Markov chain theory.

***Open problem 3:*** What is a natural algebraic framework for genomes with repeated elements, circular symmetry, and non-constant number of elements?

A large limitation of the current framework, just as with past permutation models of genomes, is that it requires the total number of genes and the number of each type of gene to remain constant. This does not allow for possible deletion events. A different model using partial permutations was proposed in (Clark et al. 2023) to study deletions. Similarly, duplications and insertions would require a more sophisticated model. Such generalizations may be possible by embedding the double coset structure into a larger semigroup or category.

## Data Availability

No data is associated with this work.
